# Improving Pulmonary Delivery of Budesonide Suspensions Nebulized with Constant-Output Vibrating Mesh Nebulizers by Using Valved Holding Chamber

**DOI:** 10.3390/pharmaceutics17060696

**Published:** 2025-05-26

**Authors:** Tomasz R. Sosnowski, Izabela Kazimierczak, Aleksandra Sawczuk, Kamil Janeczek, Andrzej Emeryk

**Affiliations:** 1 Faculty of Chemical and Process Engineering, Warsaw University of Technology, 00-645 Warsaw, Poland; izabela.kazimierczak.stud@pw.edu.pl (I.K.); aleksandra.sawczuk.stud@pw.edu.pl (A.S.); 2 Department of Allergology and Pediatrics, Medical University of Lublin, 20-093 Lublin, Poland; kamil.janeczek@umlub.pl; 3 Department of Pediatrics, Lung Diseases and Rheumatology, University Chidren’s Hospital in Lublin, 20-093 Lublin, Poland; emerykandrzej@gmail.com

**Keywords:** inhalation, nebulization, drug availability, aerosol, budesonide

## Abstract

**Background**: Vibrating mesh nebulizers (VMNs) are not only used to deliver typical pulmonary drugs but are also a promising platform for novel formulations and therapeutic applications. Typically, these devices operate continuously or on demand and are directly connected to the outflow interface (mouthpiece or mask) without valving systems that could spare the drug during exhalation. This paper examines the possibility of increasing the delivery of inhaled budesonide aerosol by attaching a valved holding chamber (VHC) to selected VMNs. **Methods**: A laboratory in vitro study was conducted for seven budesonide (BUD) nebulization products (0.25 mg/mL). The rates of aerosol delivery from VMNs alone or VMN + VHC systems were determined gravimetrically for a simulated breathing cycle, while droplet size distributions in mists were measured by laser diffraction. **Results**: The VMN + VHC systems increased the amount of aerosol available for inhalation and the fraction of fine particles that could penetrate the pulmonary region. Depending on the VMN and BUD product, a relative increase of 30–300% in the total drug delivery (*T*) and 50–350% in the pulmonary drug availability (*P*) was obtained. The results are explained by the reduction in aerosol losses during exhalation (the fugitive emission) by the VHC and the simultaneous elimination of the largest droplets due to coalescence and deposition in the chamber. Both VMN and BUD affected the aerosol’s properties and discharge mass and thus the actual benefits of the VHC. **Conclusions**: While the results confirm the superiority of VMN + VHC over VMNs alone in nebulizing BUD suspensions, they also show that it is difficult to predict the effects quantitatively without testing the individual nebulizer–chamber–drug combination.

## 1. Introduction

Budesonide (BUD) is a corticosteroid widely used to treat respiratory tract inflammation in asthma or COPD (chronic obstructive pulmonary disease) through topical administration as an inhalation aerosol [1,2]. The essential problem in drug delivery to the respiratory system is targeting certain levels of the bronchial tree, which allows the drug to act locally with minimized side effects [2,3,4]. BUD can be delivered from dry powder inhalers (DPIs), metered dose inhalers (MDIs), or nebulizers. Since it is insoluble in water, BUD formulations for nebulization (atomization) are prepared as aqueous suspensions [5,6], which must be stabilized against crystal aggregation by some ionic and surface-active additives, such as disodium edetate and polysorbate 80 [7]. Many generic BUD products for nebulization are currently available, and some of them have been studied to assess the similarity of the physicochemical properties of the formulations and atomization in a nebulizer [8]. These investigations showed that despite the identical concentration of BUD, the steroid crystals in these pharmaceutical products had different sizes. Also, physicochemical properties, such as the surface tension, conductivity, and zeta potential, were not identical, which could be responsible for the various amounts of the drug delivered to the respiratory system. This effect may be due to changes in aerosol properties and the residual volume (the volume of the suspension remaining in the device after nebulization) caused, e.g., by foaming. Recently, more attention has been given to vibrating mesh nebulizers (VMNs), which are convenient, portable, and easy-to-operate nebulizing devices [9,10]. Despite some suggestions that suspensions cannot be effectively nebulized in these devices, VMNs were proven to deliver BUD using liquid suspensions [11,12]. A critical factor is the size of active pharmaceutical ingredient (API) crystals, which must be smaller than orifices in the mesh to avoid clogging. Also, proper mesh cleaning procedures after each nebulization are crucial for VMN reliability [13].

The use of VMNs can be improved by using volumetric adapters that can hold the aerosol during exhalation [14,15,16,17]. Sarhan et al. [18] studied the salbutamol nebulization in the system, which consists of the Aerogen Solo vibrating mesh nebulizing head connected to a low-volume dedicated chamber that enables this small VMN to be conveniently used by spontaneously breathing patients (today, this system is offered as the Aerogen Ultra nebulizer [19]). They showed that using this chamber allowed for more than 60% more salbutamol delivery than from a VMN directly connected to a standard T-piece mouthpiece adapter. They also found a reduction in the mass median aerodynamic diameter (*MMAD*) of the delivered aerosol, which suggests that the holding chamber played a role in eliminating large droplets. Adding a holding chamber is essential for VMNs, which operate mainly continuously. In contrast to many jet nebulizers, they do not have any valves, allowing the aerosolized drug to be saved during the exhalation phase of breathing. The easiest way to improve delivery from VMNs is to adapt typical valved holding chambers (VHCs), also known as valved spacers, used with metered dose inhalers (MDIs). Such VHCs are recommended to reduce the effects of coordination errors and increase the dose delivered to the lungs with a simultaneous reduction in potential side effects due to aerosol deposition in the upper airways [20]. The current work focuses on a quantitative analysis of aerosol delivery during the nebulization of one original and six equivalent (generic) BUD suspensions using three different VMNs combined with a typical VHC.

## 2. Materials and Methods

### 2.1. Nabulizers and BUD Products

**Figure 1:**
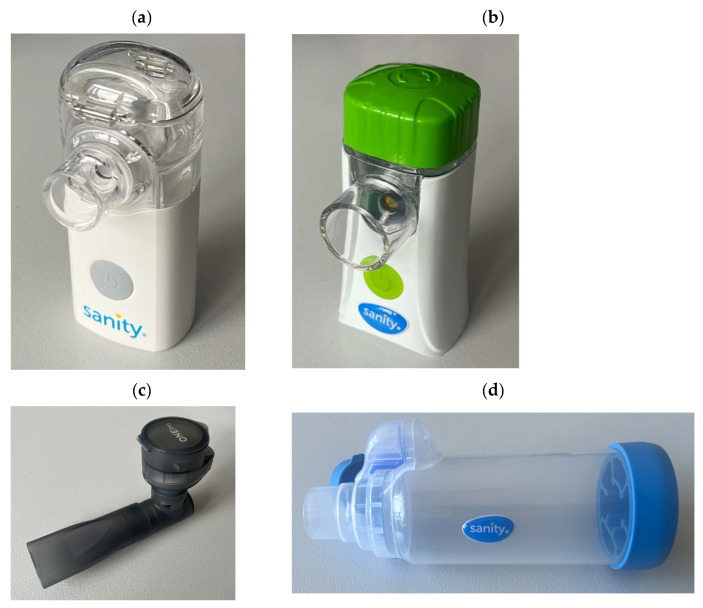
Vibrating mesh nebulizers used in the study: (**a**) Sanity Silent Mesh—SM, (**b**) Sanity Fast Mesh—FM, (**c**) Ca-Mi One Pro—OP, and (**d**) valved holding chamber Turbo Chamber Sanity.

The following VMNs were studied: Silent Mesh (SM—Sanity, Poland), Fast Mesh (FM—Sanity, Poland), and One Pro (OP—Ca Mi, Italy). A Sanity Turbo Chamber with a volume of 175 mL was used as an example of a VHC typically used with MDIs. All VMNs and the VHC are shown in Figure 1. The study was conducted on seven products of budesonide suspensions with a concentration of 0.25 mg/mL, which are listed in alphabetical order in Table 1, and which were randomly coded as B1, B2, ..., B7. The reason for said coding was to facilitate the analysis and presentation of the results without directly comparing BUD products with each other, which was not the purpose of this study and should not be performed using the methods used here. Nebulizers were filled with the product transferred from the ampoule (2 mL) with the precautions listed in the product leaflet (gentle mixing without shaking).

### 2.2. Mass Output of VMNs Operated Without and with VHC

**Figure 2:**
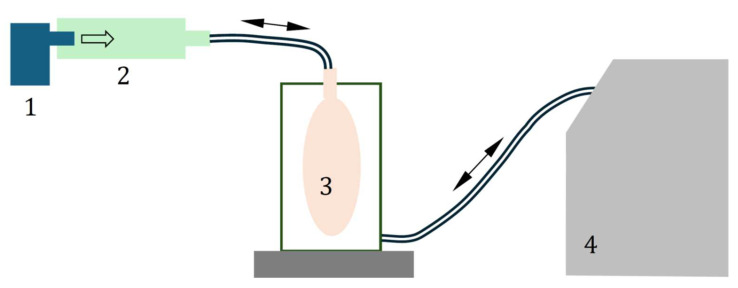
A schematic of the experimental set-up: 1—mesh nebulizer; 2—valved holding chamber (optional); 3—balloon; 4—breathing simulator ASL 5000 XL. The arrows show the bidirectional breathing airflow.

The drug release rates from nebulizers alone (without the VHC) were determined by weighing the nebulizers at 2 min intervals during aerosol emission using a sinusoidal flow simulated according to compendial recommendations [21]. In the studies of the nebulizers with the VHC, the increase in the VHC mass was also determined in the same time intervals to evaluate the amount of aerosol deposited in the chamber. The sinusoidal flow was generated by an ASL 5000XL breathing simulator (Ingmar Medical, Pittsburgh, PA, USA), adapting the breathing curve of a healthy adult. A schematic of the measurement system is shown in Figure 2. Each type of measurement was repeated three times. The proposed gravimetric method was selected as best suited for use with the breathing simulator for tested VMNs characterized by a high mass output. The use of aerosol collection on filters was ineffective due to fast filter clogging, with a rapid increase in the pressure drop and choking of the simulator.

### 2.3. Aerosol Droplet Size Distribution for VMNs Operated Without and with VHC

The droplet size distributions (DSDs) in the aerosol cloud emitted out of each system, i.e., from a nebulizer alone or with the attached VHC, were determined using a laser diffraction method (Spraytec aerosol spectrometer—Malvern Panalytical, Malvern, UK). The measurements were conducted in a droplet size range of 0.1–900 μm and allowed for the determination of the *Dv50* (or VMD—the median of the volumetric size distribution) and fine particle fraction (*FPF*—the mass fraction of droplets smaller than 5 μm). Based on the *Dv50*, *MMAD* was calculated as the parameter more commonly used than *Dv50* to characterize the quality of inhalable aerosols. It is worth noting that according to compendial recommendations [21], *MMAD* is typically determined during impactor studies by considering only droplets that penetrate beyond the impactor inlet, i.e., omitting the mass of the ballistic fraction separated in this inlet [22,23]. This type of inlet was not used in the measurements made with the diffraction spectrometer, so finding *MMAD* needed additional calculations based on the complete DSD determined by the spectrometer and assuming that the density of diluted BUD suspension was practically equal to the density of water.

### 2.4. The Calculation of the Relative Increase in the Total and Pulmonary Drug Availability

The aerosol emission rates using a nebulizer without and with the VHC were determined based on the data obtained. These were used to calculate the total drug availability, *TDA*, which considers the amount of the drug that can reach the respiratory system when the patient uses the nebulizer alone (*TDA_N_*) and with a VHC (*TDA_VHC_*). The calculations of *TDA_N_* were performed assuming that a patient inhales the aerosol only during the first 40% of the time of the whole respiratory cycle, since during pause and expiration, i.e., the remaining 60% of the time of the cycle, the aerosol produced by the nebulizer is emitted outside, as the so-called fugitive aerosol [15,24]. For nebulizers that work with VHCs, such emissions do not occur, so the whole amount of the aerosol that flows from the nebulizer to the chamber either settles down inside or flows out via the chamber mouthpiece.

To compare the effect of the VHC on the *TDA*, the relative increase in the total availability of the drug, *T* (in %), was calculated as follows:

(1) $$T=\frac{\;{TDA}_{VHC}-{TDA}_{N}}{{TDA}_{N}}\cdot 100\%=\left(\frac{\;{TDA}_{VHC}}{{TDA}_{N}}-1\right)\cdot 100\%$$

and *T* > 0 means a positive effect of using the VHC regarding the *TDA*.

Measuring the DSD in the aerosol that left a nebulizing system (nebulizer alone or nebulizer with the VHC) allowed us to determine the mass of droplets smaller than 5 μm (*FPM*—fine particle mass) and their fraction in the whole aerosol (*FPF*). The *FPF* was then used to calculate the pulmonary drug availability (*PDA*), using the following relationship:

(2) $${PDA}_{i}={FPF}_{i}\cdot {TDA}_{i}$$

where index *i* = *N* or *VHC* for systems without and with the attached VHC, respectively. By analogy to the effect of the VHC on the *TDA*, the relative increase in the pulmonary drug availability, *P* (in %), achieved when using the VHC, was calculated as follows:

(3) $$P=\frac{\;{PDA}_{VHC}-{PDA}_{N}}{{PDA}_{N}}\cdot 100\%=\left(\frac{\;{PDA}_{VHC}}{{PDA}_{N}}-1\right)\cdot 100\%$$

A positive value of *P* means that the VHC increased the *PDA*.

The differences in the values of all parameters (*FPF*, *MMAD*, *T*, and *P*) for the drugs delivered from a given nebulizer were analyzed statistically by the post hoc Tukey’s test to assess the statistical significance of differences between the results (*p* < 0.05).

## 3. Results

### 3.1. The Aerosol Mass Output of the VMNs Without and with the VHC

The results of the mass output of the VMNs, SM, FM, and OP, working without and with the VHC during nebulization of each BUD product are listed in Table 2, Table 3 and Table 4.

The mass output of each VMN working without the VHC was in the range of 0.2–0.3 g/min and slightly depended on the BUD product (SM nebulizer: 0.19–0.23 mg/min; FM nebulizer: 0.18–0.24 g/min; OP nebulizer: 0.25–0.29 g/min). The reproducibility of the results was good. After attaching the VHC, the output remained almost in the same range for the SM and OP nebulizers. Still, it visibly increased in the FM nebulizer (up to 0.42 g/min), which could have been due to the design of the outlet tube in this VMN that can reduce the mass of released aerosol from the FM nebulizer without the VHC. This will be analyzed in more detail in Section 4.

### 3.2. Fine Particle Fraction and MMAD of Nebulized BUD Products

**Figure 3:**
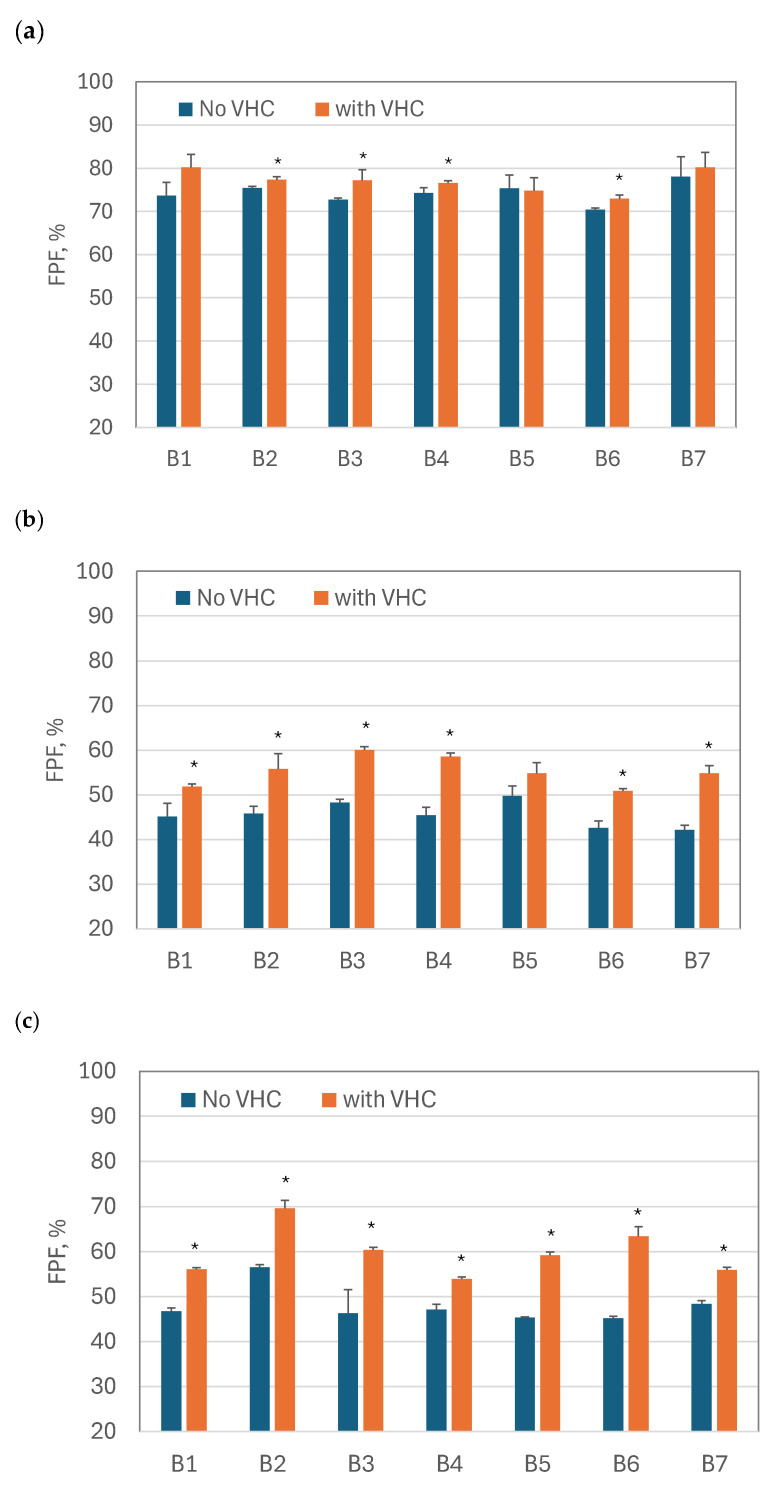
Fine particle fraction of the aerosol that leaves the nebulizers alone (‘No VHC’) and with the holding chamber (‘with VHC’): (**a**) SM nebulizer; (**b**) FM nebulizer; (**c**) OP nebulizer. Statistically significant differences are indicated by an asterisk (*p* < 0.05).

The average *FPF* values and the standard deviation (*n* = 3) for both configurations, i.e., the VMN alone and VMN + VHC, are compared for all BUD products (B1–B7) and nebulizers (SM, FM, and OP) in Figure 3. They show that *FPF* always increased when VMNs were used with the VHC. For all drugs in the nebulizer OP and for most of the medicines in the nebulizers SM and FM, the differences in *FPF* for VMNs and VMN + VHC were statistically significant (*p* < 0.05).

**Figure 4:**
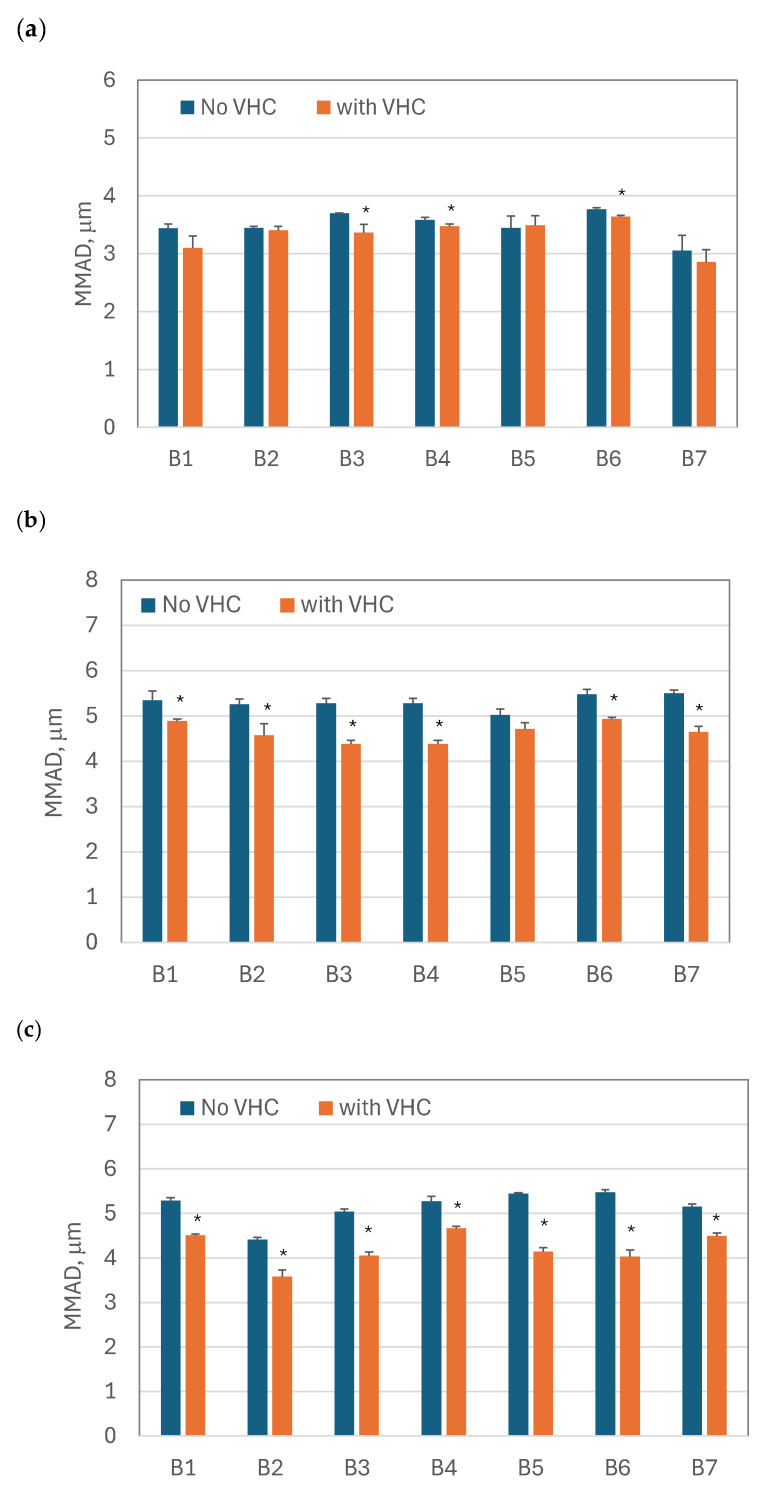
*MMAD* of the aerosol that leaves the nebulizers alone (‘No VHC’) and with the holding chamber (‘with VHC’): (**a**) SM nebulizer; (**b**) FM nebulizer; (**c**) OP nebulizer. Statistically significant differences are indicated by an asterisk (*p* < 0.05).

The average *MMAD* values and the standard deviation (*n* = 3) for both configurations, i.e., the VMNs alone and VMN + VHC, are compared for all BUD products (B1–B7) and nebulizers (SM, FM, and OP) in Figure 4. Similar to *FPF*, changes in *MMAD* were statistically significant for all BUD products nebulized in the OP nebulizer, for all except B5 in the FM nebulizer, and for the B3, B4, and B6 products in the SM nebulizer. The most substantial increase in *FPF* was observed when the nebulizer (alone) generated large droplets, i.e., when the initial *FPF* was small. This also corresponded to a larger *MMAD*. Depending on the BUD product, the *FPF* increased from 42–50% in the FM nebulizer alone to 51–60% in the FM + VHC system. Similarly, the *FPF* notably rose from 45–56% in the OP nebulizer alone to 54–70% in the OP + VHC system. In contrast, only a minor *FPF* increase was found in the SM nebulizer, from 70–78% to 73–80%.

The *FPF* changes were directly related to the results of *MMAD,* which mainly depended on the nebulizer and then on the nebulized BUD product. Regardless of the BUD sample, the original *MMAD* values (for VMNs without the VHC) were 3.1–3.8 μm in the SM nebulizer, 5.0–5.5 μm in the FM nebulizer, and 4.4–5.5 μm in the OP nebulizer. After attaching the VHC, the *MMAD* was reduced, depending on the nebulizer and BUD product. In the SM nebulizer with the VHC, the *MMAD* decreased to 2.9–3.5 μm, the FM nebulizer to 4.3–4.9 μm, and the OP nebulizer to 3.6–4.5 μm.

A significantly reduced *MMAD* in the FM and OP nebulizers with the VHC (Figure 4a and Figure 4b, respectively) and the noticeable *FPF* increase in these devices can be explained by larger droplets originally generated by these nebulizers. Since the mechanism of droplet deposition in the VHC was mainly inertial and gravitational, the largest droplets were eliminated in the chamber to a greater extent than the smaller ones. Accordingly, the aerosols that penetrated the VHC were characterized by a lower *MMAD* and a higher *FPF* (Figure 3b and Figure 3c for the FM and OP nebulizers, respectively). The results obtained in the SM nebulizer alone, characterized by a low *MMAD*, support this explanation (see Figure 3a and Figure 4a, which show only minor changes in *MMAD* and *FPF*, respectively).

### 3.3. Relative Increase in Total and Pulmonary Drug Availability

**Figure 5:**
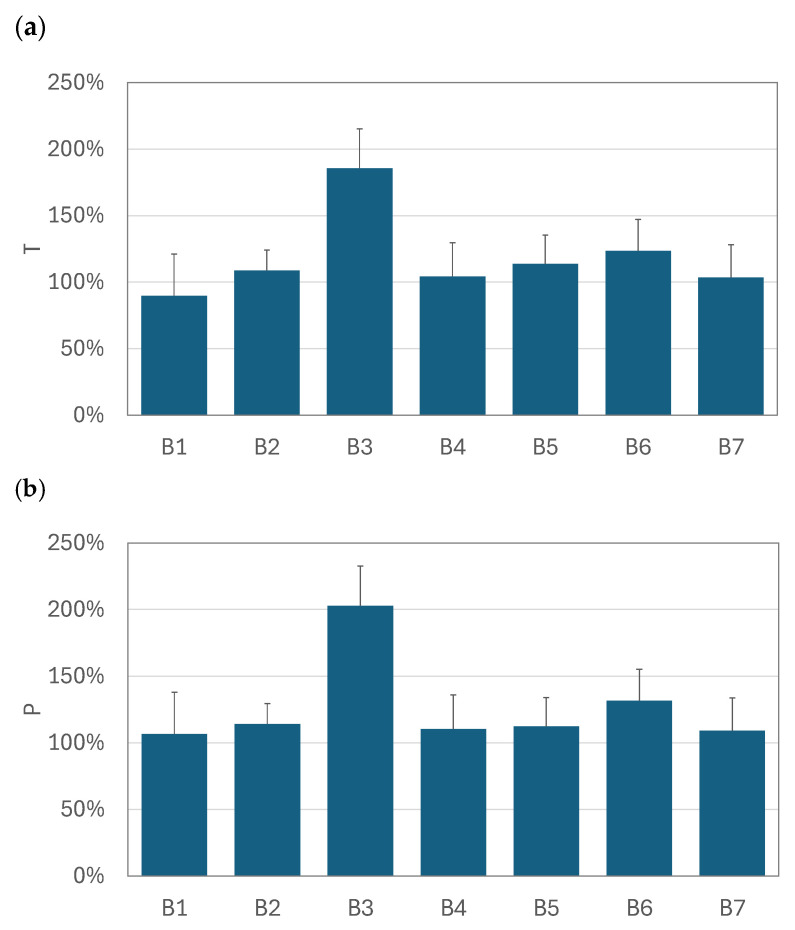
The relative increase in (**a**) total availability (*T*) and (**b**) pulmonary availability (*P*) of aerosols administered by SM + VHC. Error bars denote standard deviation (*n* = 3).

**Figure 6:**
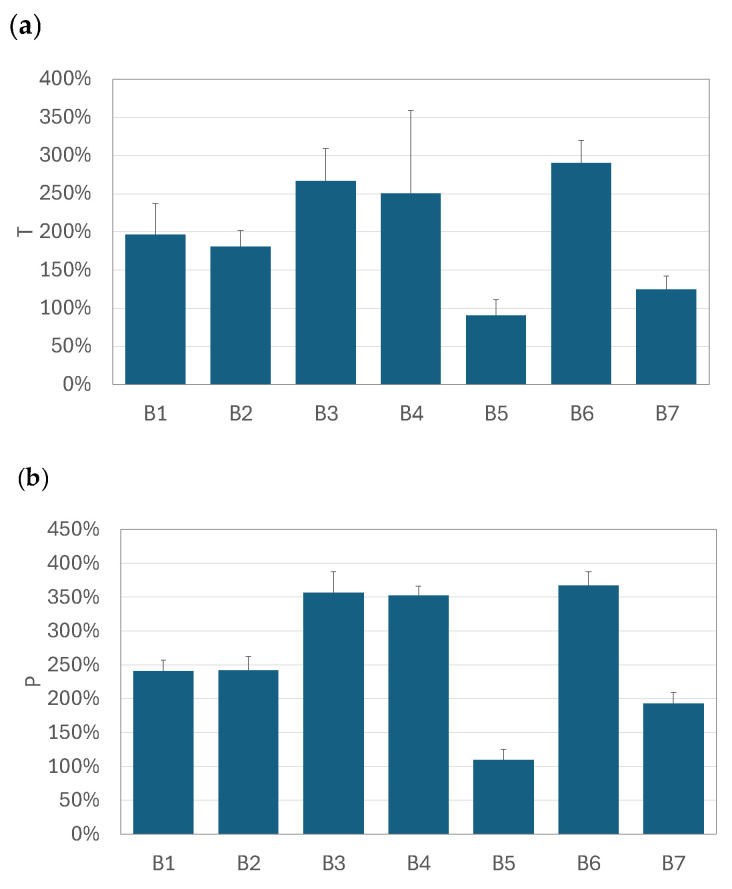
The relative increase in (**a**) total availability (*T*) and (**b**) pulmonary availability (*P*) of aerosols administered by FM + VHC. Error bars denote standard deviation (*n* = 3).

**Figure 7:**
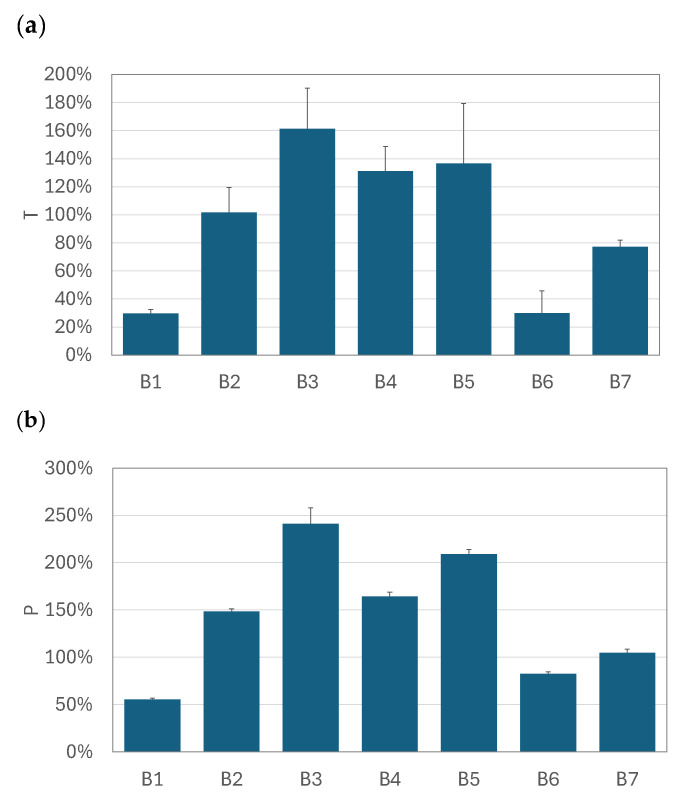
The relative increase in (**a**) total availability (*T*) and (**b**) pulmonary availability (*P*) of aerosols administered by OP + VHC. Error bars denote standard deviation (*n* = 3).

The graphs in Figure 5, Figure 6 and Figure 7 compare the relative increase in the total drug availability (*T*) and the pulmonary availability (*P*) for each drug–nebulizer combination. The *T* and *P* values were always positive, which confirms that using the VHC was beneficial for the total (*TDA*) and pulmonary drug availability (*PDA*) in all VMNs studied. It was also seen that the relative increase in drug availability depended not only on the VMN but also on the nebulized BUD product. In the SM nebulizer (Figure 5), *T* and *P* were approximately 90–130% for all products except for B3, where both parameters were 180–200%. The *T* and *P* values obtained in the OP nebulizer (Figure 7) were close to those in the SM device, i.e., *T* was below 160%, *P* was below 250%, and both parameters depended on the nebulized BUD product. In some cases (products B1 and B6), *T* was as low as 25% (but still positive), resulting in *P* values as low as 50–75%. These values were smaller than the minimum values obtained in the SM nebulizer (90–100%). The highest *P* values (110–370%) were found in the FM nebulizer (Figure 6), suggesting a strong increase in the pulmonary availability of the aerosolized drugs with the VHC. The maximum increase was observed for drugs B3, B4, and B6. There was a visible difference between the range of *T* (290% at the maximum) and *P* (above 360% at the maximum), suggesting a significant role of the VHC in removing the largest droplets by the mechanisms suggested earlier.

## 4. Discussion

Nebulization is a method of drug atomization commonly used in the pulmonary delivery of drugs by inhalation. It does not require the full cooperation of a patient, since the aerosolized medicine is administered during unforced (tidal volume) breathing. Therefore, this method is very convenient for drug delivery to children, elderly patients, people with disabilities, or even unconscious patients. Nebulization is also preferentially used in developing new drugs or drug vehicles for aerosol therapy. Still, it must be ensured that atomization to fine droplets does not compromise their biological activity [25,26]. Nebulizers are promising drug-delivery devices for the pulmonary delivery of controlled-release formulations [27]. However, the most common applications are related to delivering anti-inflammatory drugs (corticosteroids) or β2-mimetics. Among the different nebulizing devices, vibrating mesh nebulizers have become very popular due to their portability, silent operation, and narrower droplet size distribution, which allows for more precise drug targeting in the diseased areas of the respiratory system [28,29]. In contrast to jet nebulizers, most VMNs do not have valves that can prevent aerosol outflow from the device during exhalation. This results in significant aerosol losses during the oscillatory flow of breathing. The attachment of a valved holding chamber allows for the storage of the emitted aerosol during the exhalation phase of the breathing cycle. The advantages of VHCs with VMNs over jet nebulizers concerning the delivered dose to the lungs have already been demonstrated in several in vitro and in vivo studies [14,18]. However, none of these investigations have compared the VMN operation alone and after the connection of typical VHCs adapted from MDIs. Also, no study has focused on the nebulization of different BUD formulations (originator vs. generics) in such systems.

The results of this study firstly show that BUD suspensions can be nebulized by VMNs for a long time without mesh clogging. This was confirmed by the reproducible results of aerosol emission for all drugs at various nebulizer lifetimes without a drop in the aerosol mass output. The literature data suggest that BUD crystals in the nebulization suspensions (both the original and generic) are typically smaller than 3 μm [8], which means that they can pass the mesh. Although the properties of the mesh of the VMNs studied in this work are not known (data are not provided by the manufacturers, and they cannot be measured without destroying the devices), it is expected that all these devices have a similar, common design (aperture diameter of ~3 μm [8,30,31])

A more significant finding was that the total mass of the aerosol and the mass of the fine particles increased when any of the three VMNs tested were used together with the VHC. This also means that VMNs operating without a VHC can deliver much lower aerosol doses than expected because of the periodic inhalation during a constant drug release from the nebulizer. Attaching a VHC to VMNs allows the drugs to be delivered similarly to valved jet nebulizers but additionally reduces the fraction of coarse droplets, which are not needed for the treatment of lower airways. Changes in drug delivery from the VMNs after using the VHC were quantitatively assessed by two parameters: the relative increase in the total availability of the drug, *T* (Equation (1)), and the relative increase in the pulmonary drug availability, *P* (Equation (3)). Both *T* and *P* were always positive (*T* = 30–300% and *P* = 50–350%, depending on the nebulizer and BUD product), which confirmed the benefits of the VHC. Differences in the *T* and *P* for products B1–B7 nebulized in a single VMN can be explained by the variability in the physicochemical properties of the BUD suspensions. The literature data show that despite the same steroid mass, such formulations differ in surface tension, crystal size, and crystal agglomeration [8].

**Figure 8:**
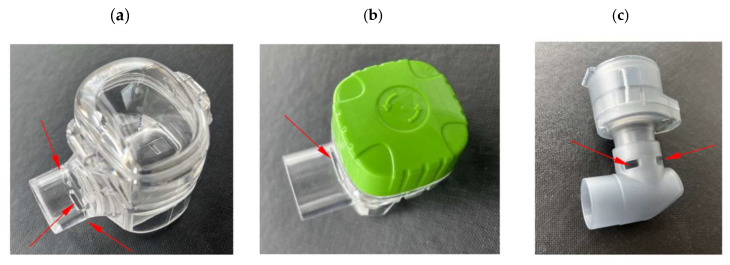
Arrangement of the air inlets in the outlet tubes of the VMNs (air inlets are indictaed by arrows): (**a**) three inlets in the SM; (**b**) single top inlet in the FM; (**c**) four inlets in the OP elbow.

It is also interesting to note that for the same drug, *T* and *P* can be high in one VMN but low in another device. For instance, the delivery of the B6 aerosol was strongly enhanced by the VHC in the FM nebulizer (*T ≈* 300%; *P ≈* 350%) but not in the SM nebulizer (*T ≈* 120%; *P ≈* 130%) or the OP nebulizer, where the gain from using the VHC was relatively low for the same B6 product (T *≈* 30%; P *≈* 80%) compared to the other BUD products (*T* up to 160% and *P* up to 240%). This clearly shows that the choice of VMN is essential. Variability can be caused by differences in the material and structure of the mesh [32], as well as other design features of the device. For instance, the number and arrangement of air inlets in the VMNs’ outlet tubes are different in the VMNs studied, as shown in Figure 8. The air inlet in the FM nebulizer is located above the mesh, so the liquid condensed from the aerosol during exhalation collects on the lower surface of the tube. This explains why the measured output rate of the FM nebulizer without the VHC was low and significantly increased after attaching the chamber, which prevented liquid collection in the outlet tube (Table 3). This differs from the SM nebulizer, where the collected liquid can drain through holes in the tube. In contrast to these two nebulizers, the OP nebulizer has a different configuration—the mesh is horizontal, so the aerosol is pushed down through the elbow element, which redirects the aerosol to flow horizontally through the mouthpiece. Large droplets are preferentially deposited in this bend, so they cannot be emitted, reducing the total aerosol mass output.

**Figure 9:**
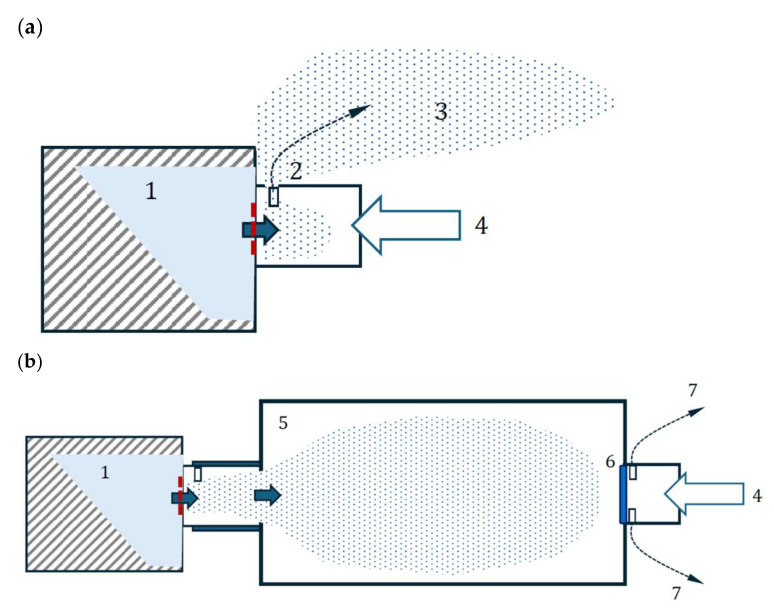
Comparison of VMN operation during air exhalation by a patient: (**a**) VMN alone; (**b**) VMN with VHC. 1—nebulized drug in a VMN head; 2—opening in nebulizer outflow tube; 3—fugitive aerosol; 4—airflow exhaled by a patient; 5—valved holding chamber; 6—unidirectional valve; 7—exhaled air released from the system.

The principles of flow modification by the VHC for the aerosol and exhaled air are depicted in Figure 9. It is shown that the aerosol was pushed outside as a fugitive aerosol when the VMN worked without the VHC (Figure 9a). In contrast, when the VHC was used, the aerosol collected inside the chamber (with possible losses due to deposition on the walls), and no fugitive aerosol was released. In this case, the exhaled air was allowed to flow out through a unidirectional valve mounted at the mouthpiece tube of the VHC, i.e., without mixing with the aerosol in the chamber. These differences in system operations were responsible for the main benefits of using the VHC with the VMNs. Despite the increased pulmonary aerosol availability, a reduced aerosol deposition in the upper airways was expected due to an increase in *FPF*. This is an essential issue in the safety of BUD inhalation.

A limitation of this study is that all measurements were performed gravimetrically, i.e., without chemical detection of the BUD dose in the aerosol. This may have led to overestimations of FPF, considering that not all small aerosol droplets contain BUD due to size limitations (BUD crystals must be smaller than the droplet diameter to be inside the droplet). Nevertheless, the data presented allow us to demonstrate a notable quantitative difference in the emission rate and aerosol properties of the nebulized BUD suspensions. The literature data show differences in the BUD content in the emitted aerosol as low as 10–15% [8], whereas the increase in the mass of the aerosol delivered from the VMN + VHC systems shown here is even 3-fold. Therefore, this solution can be considered potentially relevant to clinical outcomes, although future work focused on the actual BUD dose obtained in the proposed nebulizer system is needed.

## 5. Conclusions

The in vitro investigations presented in this work confirm that using a VHC as an add-on device to VMNs is always beneficial regarding both the effectiveness of pulmonary delivery and the safety of the inhalation of nebulized budesonide suspensions. A VHC always causes partial losses of the aerosolized drug due to droplet deposition in the chamber, but simultaneously, it holds the aerosol during exhalation, eliminating aerosol emission to the surroundings. Since the losses are caused mainly by impaction and sedimentation mechanisms, only the largest droplets (>5 μm) are captured in the VHC. This increases the number of inhaled aerosols that can reach the distal airways, i.e., the target of BUD delivery. With a VHC, unwanted drug accumulation in the upper respiratory tract is also reduced. Due to temporary aerosol accumulation in the chamber, the total amount of drug that can be inhaled is increased compared to a nebulizer without a chamber when more than 50% of the released drug is spread as a fugitive aerosol. Therefore, using a VHC with the standard constant-output non-valved vibrating mesh nebulizers is recommended.

The results of these studies also show that the increase in the inhaled aerosol depends on the VMN model and the BUD product. This can be explained by both differences in the physicochemical characteristics of the products and the nebulizer designs, suggesting that the actual benefit of using a holding chamber with the vibrating mesh nebulizer always requires a quantitative evaluation of the specific VMN + VHC system with a given nebulization product. It was also confirmed that BUD can be nebulized in VMNs for a long period without causing mesh clogging.

## Figures and Tables

**Table 1:** BUD nebulization products (suspensions), coded as B1–B7 in random order.

BUD Product	Manufacturer (Manufacturing Authorization Holder)
BDS N, 0.25 mg/mL	Aurovitas Pharma Polska, Warsaw, Poland
Benodil, 0.25 mg/mL	Polpharma SA, Starogard Gdański, Poland
Budixon NEB, 0.25 mg/mL	Adamed Pharma, Pieńków, Poland
Nebbud, 0.25 mg/mL	Teva Pharmaceuticals Polska, Warsaw, Poland
Ondemet, 0.25 mg/mL	Zentiva Polska, Warsaw, Poland
Pulmicort, 0.25 mg/mL	AstraZeneca Pharma Poland, Warsaw, Poland
Resbud, 0.5 mg/2 mL	Lek-AM Sp. z o.o., Zakroczym, Poland

**Table 2:** Mass output (g/min) of the SM nebulizer without or with the VHC.

BUD	No VHC	SD	With VHC	SD
B1	0.217	0.043	0.201	0.034
B2	0.202	0.025	0.214	0.030
B3	0.187	0.030	0.227	0.013
B4	0.188	0.010	0.173	0.001
B5	0.224	0.026	0.227	0.028
B6	0.230	0.044	0.219	0.028
B7	0.219	0.011	0.196	0.003

**Table 3:** Mass output (g/min) of the FM nebulizer without or with the VHC.

BUD	No VHC	SD	With VHC	SD
B1	0.191	0.017	0.361	0.032
B2	0.179	0.019	0.368	0.021
B3	0.187	0.030	0.359	0.009
B4	0.216	0.014	0.406	0.011
B5	0.243	0.057	0.383	0.006
B6	0.212	0.021	0.416	0.057
B7	0.214	0.031	0.368	0.056

**Table 4:** Mass output (g/min) of the OP nebulizer without or with the VHC.

BUD	No VHC	SD	With VHC	SD
B1	0.269	0.010	0.209	0.020
B2	0.279	0.003	0.260	0.034
B3	0.263	0.007	0.324	0.039
B4	0.246	0.008	0.265	0.051
B5	0.271	0.010	0.291	0.021
B6	0.289	0.006	0.167	0.034
B7	0.279	0.015	0.258	0.003

## Data Availability

The data presented in the study are included in the article. Further inquiries can be directed at the corresponding author.

## Abbreviations

The following abbreviations are used in this manuscript:

API	Active pharmaceutical ingredient
BUD	Budesonide
COPD	Chronic obstructive pulmonary disease
DPI	Dry powder inhaler
DSD	Droplet size distribution
Dv50	Median diameter of volumetric DSD, μm
FM	Fast Mesh (nebulizer)
FPF	Fine particle fraction, %
FPM	Fine particle mass, mg
MDI	Metered dose inhaler
MMAD	Mass median aerodynamic diameter, μm
OP	One Pro (nebulizer)
P	Relative increase in pulmonary drug availability, %
PDA	Pulmonary drug availability, mg/min
SM	Silent Mesh (nebulizer)
T	Relative increase in total drug availability, %
TDA	Total drug availability, mg/min
VHC	Valved holding chamber
VMN	Vibrating mesh nebulizer
